# Simultaneous Use of Dual Bronchoscopes for Targeted Biopsy of Peripheral Lung Lesions: The Kissing Probe Technique

**DOI:** 10.3390/jcm14186425

**Published:** 2025-09-12

**Authors:** Sammy Onyancha, Njuxhersa Maloku, Isabelle Dettmer, Gernot Rohde

**Affiliations:** 1 Department of Pulmonology, St. Elisabethen Krankenhaus, 60596 Frankfurt, Germany; 2 Department of Respiratory Medicine, Medical Clinic I, University Hospital, Goethe University Frankfurt, 60596 Frankfurt, Germany

**Keywords:** PPL, peripheral pulmonary lesions, single-use bronchoscope, kissing probe technique

## Abstract

**Background:** Peripheral pulmonary lesions (PPLs) are increasingly detected due to widespread use of cross-sectional imaging and lung cancer screening. While cone-beam CT and robotic bronchoscopy have improved diagnostic accuracy, they remain resource-intensive and inaccessible in many settings. A novel technique employing simultaneous use of two bronchoscopes referred to as the “Kissing Probe Technique” was developed to provide real-time lesion localization and precise sampling using standard equipment. **Methods:** This retrospective, single-centre study included 43 patients with radiologically confirmed PPLs suspicious for malignancy. Under general anaesthesia with rigid bronchoscopy or continuous sedation with endotracheal intubation, two single-use bronchoscopes were introduced in parallel. The first (standard diameter) housed a radial EBUS probe for lesion localization, while the second (ultra-thin) guided a 1.1 mm cryoprobe to the lesion based on direct ultrasound and fluoroscopic confirmation. Cryobiopsies were performed once “kissing contact” between the radial probe and cryoprobe was established. **Results:** A total of 43 procedures were completed without major complications. The mean lesion size was 24.6 mm. Radial probe localization was successful in 86% of cases, and tool-contact confirmation was achieved in 35/43 patients (81%). The overall diagnostic yield was 83.7% (36/43). Bleeding occurred in 23% of cases and was managed conservatively without the need for escalation of care. No pneumothorax or equipment-related damage occurred. **Conclusions:** The “Kissing Probe Technique” is a safe and feasible approach for bronchoscopic sampling of PPLs. It offers a cost-effective alternative for real-time tool-in-lesion confirmation using widely available equipment. Further multicentre validation is warranted to confirm generalizability and cost-effectiveness.

## 1. Introduction

Peripheral pulmonary lesions (PPLs), defined as focal opacities within the lung parenchyma that are not directly visible during routine bronchoscopy, are increasingly encountered due to widespread use of cross-sectional imaging and lung cancer screening programs [1]. Accurate tissue sampling of these lesions is vital for early diagnosis and appropriate treatment planning, particularly in suspected lung cancer where histological subtyping and molecular profiling are mandatory.

Despite advances in bronchoscopic navigation techniques including electromagnetic navigation bronchoscopy (ENB), virtual bronchoscopy, augmented fluoroscopy, and robotic-assisted platforms the diagnostic yield of conventional bronchoscopic approaches for PPLs remains limited, often ranging between 40–70% depending on lesion size, location, and visibility [2]. A crucial factor in these limitations is the inability to ensure that biopsy tools are precisely within the lesion so-called “tool-in-lesion” confirmation.

Cone-beam computed tomography (CBCT) has significantly enhanced tool-in-lesion verification and biopsy accuracy in many centres [3,4]. However, the widespread adoption of CBCT-guided bronchoscopy is restricted by the high cost of equipment, the need for specialized infrastructure and staffing, as well as the requirement for general anaesthesia and controlled ventilation. This has prompted the exploration of more accessible, cost-effective alternatives.

One such innovative strategy involves the use of two bronchoscopes simultaneously, a concept previously applied in transbronchial cryobiopsy for interstitial lung disease, where a second bronchoscope enables rapid bleeding control [5]. Adapting this dual-scope concept to the peripheral lung lesion context allows for a dynamic, image-guided approach: one bronchoscope (standard-sized) localizes the lesion using radial probe endobronchial ultrasound (RP-EBUS), while the second (ultra-thin) concurrently delivers a cryoprobe to the exact site for sampling.

This study presents our results of utilizing the dual bronchoscope “Kissing Probe Technique” for the biopsy of peripheral lung lesions. We aim to demonstrate the feasibility, safety, and diagnostic yield of this approach and propose its utility as an affordable, adaptable tool-in-lesion strategy for institutions without access to CBCT or advanced navigation platforms.

## 2. Methods

### 2.1. Study Design and Setting

This was a retrospective, single-centre study conducted at the Department of Pulmonology, St. Elisabethen Krankenhaus, Frankfurt, Germany, between July 2024 and January 2025. The aim was to evaluate the safety, feasibility, and diagnostic yield of a novel dual-bronchoscope technique termed the “Kissing Probe Technique” for sampling PPLs. Ethical approval was obtained from the institutional ethics committee and all patients provided written informed consent.

### 2.2. Patient Selection

Patients were enrolled if they met the following inclusion criteria:

- Age ≥ 18 years;
- Presence of a radiologically confirmed peripheral lung lesion suspicious for malignancy (based on CT);
- Clinical indication for tissue biopsy for diagnostic clarification.

Exclusion criteria included:

- Uncorrectable coagulopathy (platelets < 50,000/µL or INR > 1.5);
- Severe hypoxemia;
- Hemodynamic instability.

### 2.3. Pre-Procedural Planning

All patients had undergone high-resolution chest CT prior to the planned procedure. Lesion characteristics including size, location, and bronchus sign were documented. Using virtual bronchoscopy and bronchial branch tracing a bronchoscopic route to the lesion was mapped in advance.

### 2.4. Operators, Anaesthesia, and Airway Management

**Figure 1:**
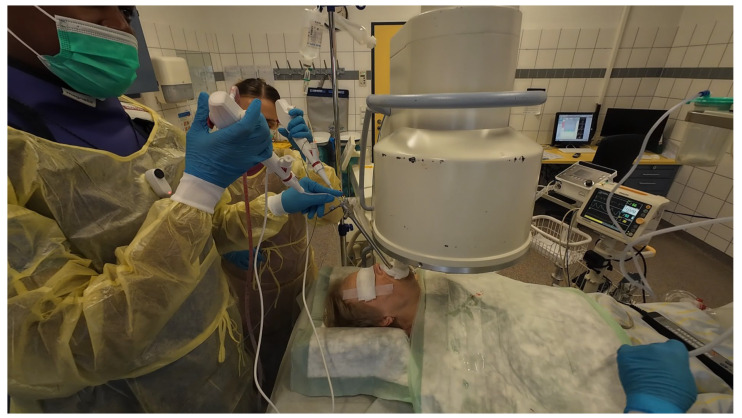
Dual operators performing “kissing probe” biopsy technique under rigid bronchoscopy.

**Figure 2:**
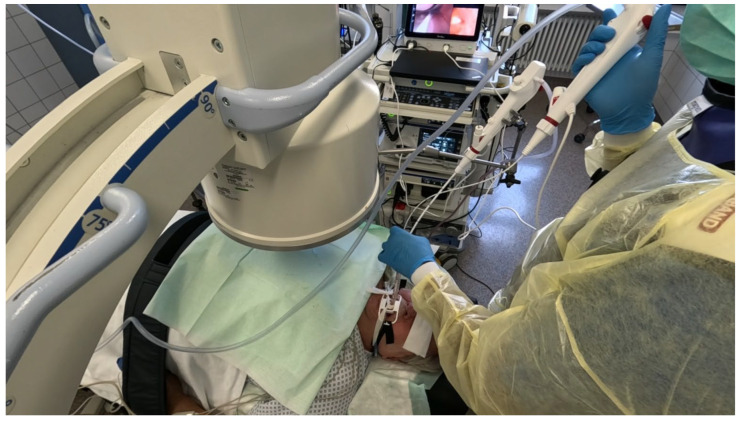
Single operator performing “kissing probe” biopsy technique over endotracheal tube.

Procedures were performed either via rigid bronchoscopy under general anaesthesia with jet ventilation or as flexible bronchoscopy under conscious sedation with spontaneous breathing. A 12 mm rigid tracheoscope (Karl Storz, Tuttlingen, Germany) was used to accommodate simultaneous passage of two bronchoscopes in the cases of rigid bronchoscopy, whilst a 9 mm endotracheal tube (Teleflex, Philadelphia, PA, USA) was implemented in the cases performed with flexible bronchoscopy. Depending on availability either two endoscopists performed the procedure (23 out of 43 cases) [Figure 1] [Video S2] or one endoscopist performed the procedure with the aid of a bronchoscope holder replacing a second operator (20 out of 43 cases) [Figure 2] [Video S1]. Patients were positioned supine and fluoroscopic guidance was used for the biopsies.

### 2.5. Bronchoscopic Technique

Two single-use video bronchoscopes from the Ambu^®^ aScope™ 5 platform were employed:

- Scope A (Standard-sized): Outer diameter 5.0 mm, 2.2 mm working channel; used for RP-EBUS guidance;
- Scope B (Ultra-thin): Outer diameter 2.7 mm, 1.2 mm working channel; used for cryobiopsy.

**Figure 3:**
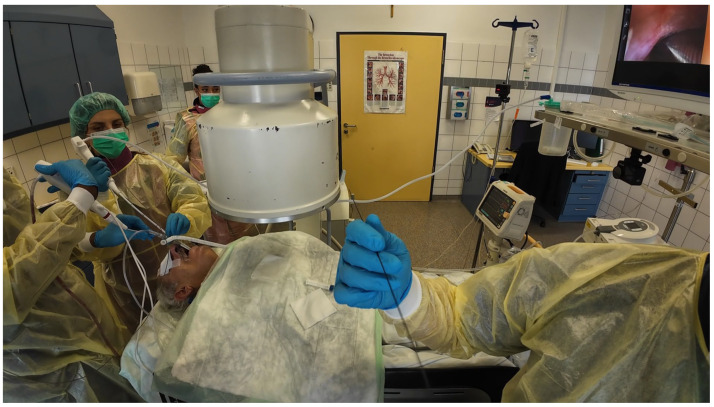
Insertion and navigation of second ultrathin bronchoscope parallel to standard bronchoscope.

**Figure 4:**
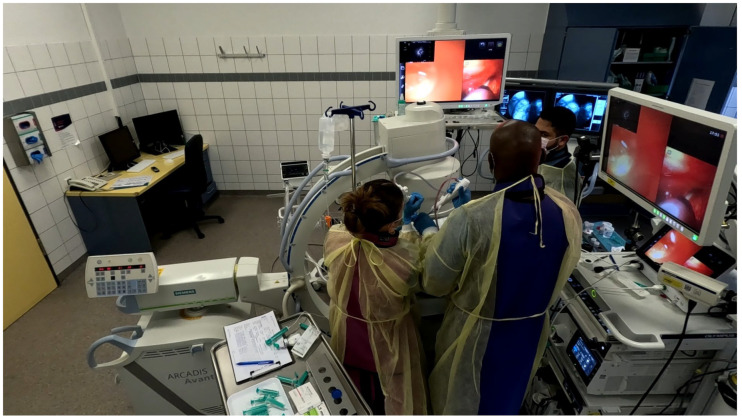
Navigation of second ultrathin bronchoscope along the radial probe path.

**Figure 5:**
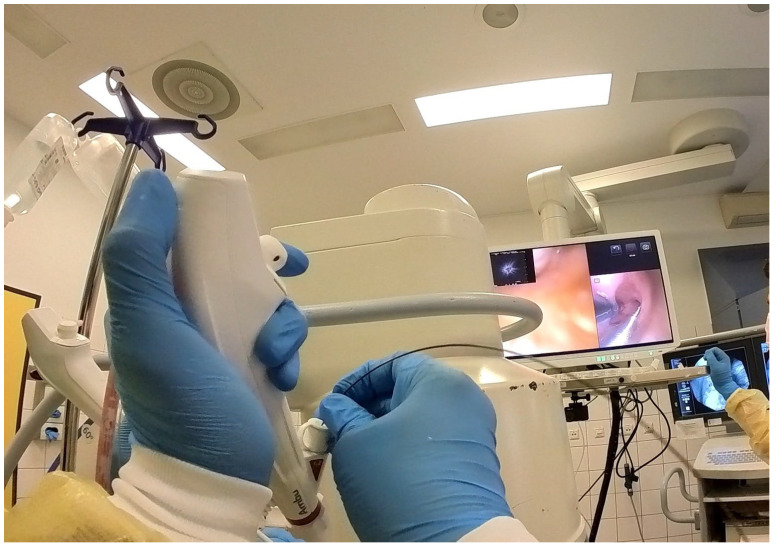
Cryoprobe advancement over ultrathin bronchoscope.

**Figure 6:**
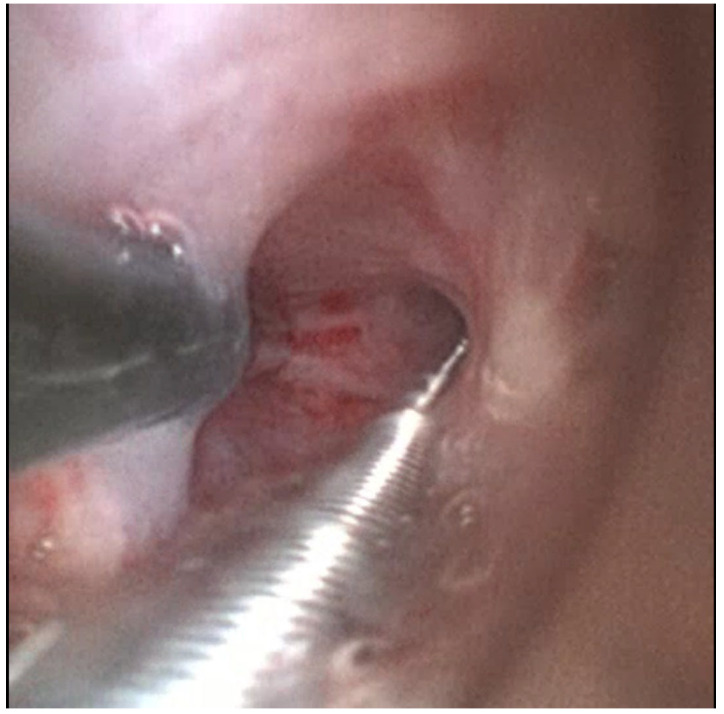
Cryoprobe placement towards radial probe tip.

**Figure 7:**
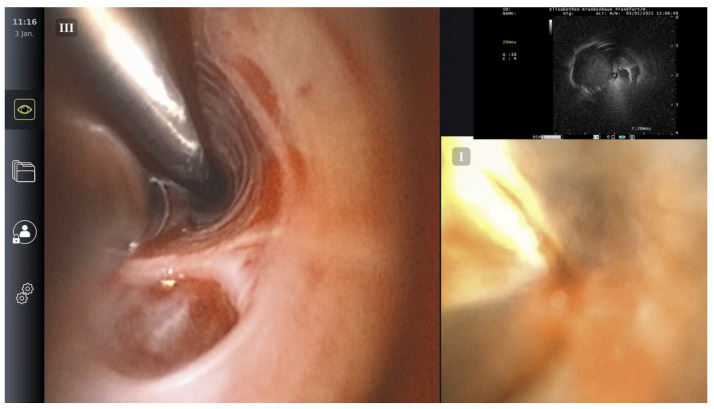
Contact between cryoprobe and radial probe observed over split screen mode.

Step-by-step Procedure:

a. Initial Navigation and Localization:

Scope A was introduced first and navigated toward the lesion using the preplanned bronchial path. A radial probe (UM-S20-17S, Olympus, Tokyo, Japan) was inserted through Scope A. The lesion was localized under real-time RP-EBUS imaging and fluoroscopy.

b. Introduction of Second Bronchoscope:

Scope B was then inserted alongside Scope A through the same endotracheal tube or rigid tracheoscope. This bronchoscope was navigated to the same segmental bronchus, following Scope A’s visual cues and fluoroscopic path [Figure 3 and Figure 4].

c. Kissing Probe Confirmation:

A 1.1 mm cryoprobe (Erbecryo 20402-401, Erbe Elektromedizin, Tübingen, Germany) was advanced through Scope B to the depth corresponding to the radial probe tip [Figure 5 and Figure 6]. “Kissing contact” was confirmed by observing distortion or artifacts on the EBUS image caused by the cryoprobe tip [Figure 7]. Fluoroscopy was used for additional depth confirmation and probe contact was confirmed in at least two perpendicular fluoroscopic views.

d. Cryobiopsy:

After withdrawing the radial probe to prevent damage, the cryoprobe was activated (freeze time: 3–4 s), and the specimen was retrieved en bloc with Scope B. Up to 5 cryobiopsies were obtained per patient.

e. Post-biopsy Management:

Scope A remained in position to allow immediate inspection of the biopsy site. Bleeding was controlled using suction, cold saline, and wedging of Scope A’s tip. If required, topical epinephrine or Fogarty balloon occlusion was available.

### 2.6. Post-Procedural Monitoring

All patients received post-procedural chest radiography to exclude pneumothorax. They remained admitted in the pulmonology unit overnight and were released the next day.

### 2.7. Histopathology and Diagnostic Criteria

Tissue specimens were processed using standard histology techniques. Diagnostic adequacy was defined as sufficient material for histological diagnosis, including immunohistochemistry (IHC) and molecular testing (e.g., Epidermal Growth Factor Receptor-EGFR, Anaplastic Lymphom Kinase-ALK) in suspected malignancies.

A conclusive diagnosis was categorized as:

- Malignant (specific tumour type identified);
- Specific benign diagnosis (e.g., organizing pneumonia, granulomatous disease);
- Nondiagnostic (insufficient or nonspecific histological features).

### 2.8. Outcome Measures

Primary outcome:

- Diagnostic yield (conclusive histological diagnosis from cryobiopsy).
- Secondary outcomes:
- Number of diagnostic samples obtained;
- Complications (bleeding, pneumothorax, need for interventional management, e.g., chest drain placement);
- Procedure duration (from scope insertion to final biopsy).

## 3. Results

A total of 43 patients underwent bronchoscopy using the dual bronchoscope technique. The mean age was 64.3 years (range: 38–82). Patients with smoking history accounted for a majority of the cohort with 21 current smokers (48.8%) and 15 former smokers (34.9%). Patient characteristics are summarized in Table 1.

The mean lesion size was 24.6 mm (range: 10–40 mm). Most lesions were located in the upper lobes: right upper lobe (RUL) in 46.5% (20/43) and left upper lobe (LUL) in 23.3% (10/43). Right lower lobe (RLL) and left lower lobe (LLL) lesions accounted for 30.2% of cases.

A positive bronchus sign on pre-procedure CT was present in 83.7% (36/43) of patients, suggesting favourable endobronchial access.

All 43 procedures were successfully completed without need for conversion or premature termination. The mean procedure duration was 42 min for all cases. Dual-operator cases had a shorter average procedure duration of 39 min while the single-operator cases had an average duration of 46 min. Chronological review of the cases showed reduced procedural time in later cases as compared to the initial cases, with plateaued procedural times after the seventh case, suggesting a learning curve effect.

A mean of three cryobiopsies (range: 2–5) were obtained per patient.

Lesion localization using RP-EBUS was achieved in 86% (37/43) of patients. Of these, 53.5% (23/43) showed a clear concentric ultrasound signal and 32.5% (14/43) had eccentric signal. In six patients (14%), initial EBUS localization failed, and the cryoprobe was advanced based solely on CT mapping and fluoroscopic feedback.

Tool-contact confirmation was defined as visible interaction between the radial probe and cryoprobe on ultrasound imaging with fluoroscopic confirmation. This was achieved in 81.4% (35/43) of the cases. Key procedural metrics are outlined in Table 2.

Of the 43 patients, 36 received a definitive histological diagnosis, yielding a diagnostic rate of 83.7%. In the subgroup analysis, a diagnostic yield of 82.6% (19/23) was observed in the dual-operator cases whilst a diagnostic yield of 85.0% (17/20) was noted in single-operator cases using a scope holder.

Malignant diagnoses were made in 30 patients (69.8%), including adenocarcinoma in 18 cases, squamous cell carcinoma in 9 cases, small cell carcinoma in 2 cases and atypical carcinoid in 1 case.

Benign diagnoses were established in six patients (14.0%), including four cases of organizing pneumonia and two cases of tuberculosis.

In seven patients (16.3%), the cryobiopsies were non-diagnostic due to non-specific inflammatory changes. Among these non-diagnostic cases, most were associated with challenging anatomical features; five lacked a bronchus sign on CT, four were located in the lower lobes, and three demonstrated eccentric radial EBUS signals. These findings suggest that lesion characteristics strongly influence diagnostic success and should guide patient selection and technique refinement. Final histological results are summarized in Table 3.

Among the 30 malignant cases, adequate tissue for complete immunohistochemistry (IHC) and molecular testing was available in 25 cases (83.3%).

There were no major complications or need for escalation of care. Minor adverse events included minor bleeding in nine patients and moderate bleeding in one patient. Complication analysis revealed that minor bleeding was more common in lesions >25 mm and in cases where ≥4 cryobiopsies were performed, suggesting both lesion size and sampling intensity may influence bleeding risk. No significant association was found between lesion location and complication rate. There was no need for chest drain placement, and no procedure-related mortality. The complications are summarized in Table 4.

## 4. Discussion

This study explores the feasibility, diagnostic performance, and safety of the simultaneous dual-bronchoscope technique for the biopsy of peripheral pulmonary lesions. Our findings demonstrate that this approach is not only technically feasible but also yields high diagnostic accuracy (83.7%) while maintaining a favourable safety profile.

Traditional transbronchial forceps biopsy techniques often suffer from limited reach and inadequate sampling, particularly in peripheral lesions located beyond the segmental bronchi. While advanced bronchoscopic navigation modalities like ENB, robotic bronchoscopy, and CBCT have been developed to address these limitations [6,7], they are not universally accessible. CBCT provides unparalleled tool-in-lesion confirmation [8,9,10] but requires substantial capital investment, specialized staffing, and often general anaesthesia with controlled ventilation, factors that restrict its routine use in many centres.

Across guidance platforms, pooled diagnostic yields for navigation bronchoscopy generally cluster around 70–80% (ENB/RP-EBUS/virtual bronchoscopy), with higher yields when real-time tool-in-lesion imaging added [11,12,13]. Meta-analyses report around 71–75% pooled yields for guided bronchoscopy overall, and around 85% for combined CBCT + r-EBUS, while r-EBUS alone varies with lesion features and probe position. Our cohort’s 83.7% yield with real-time ”kissing contact” is therefore comparable to CBCT-assisted approaches despite relying only on standard bronchoscopic equipment.

In the subgroup analysis, a favourable diagnostic yield was recorded in dual-operator cases (82.6%) as well as in single-operator cases (85%), with no difference in complication rates. This suggests both approaches are feasible although dual-operator cases had a shorter procedure time on average. Therefore, the single-operator approach may be advantageous where staffing is limited, while the dual-operator may allow for a smoother workflow in complex cases.

### 4.1. Advantages of the Dual-Bronchoscope Technique

a. Improved Targeting Accuracy:

The core innovation in our method lies in the real-time pairing of a radial probe and a cryoprobe at the same anatomical location. By visualizing the physical “kiss” between the two instruments, the operator gains immediate confirmation that the biopsy tool is at the correct site. This direct feedback minimizes sampling error, a major limitation in PPL biopsy.

b. Superior Sample Quality:

Cryobiopsy has been shown to retrieve larger and better-preserved tissue specimens compared to forceps biopsy [14]. In our study, tissue adequacy for histological, immunohistochemical, and molecular testing was achieved in 83.7% of cases. This is particularly relevant in the era of precision oncology, where PD-L1 expression and genomic mutation panels are necessary to guide targeted therapies.

c. Enhanced Safety Through Redundancy:

Having a second bronchoscope in place during biopsy allowed for immediate access to the bleeding site. In our series, all mild-to-moderate bleeding episodes were successfully controlled through suction, cold saline, and positional tamponade, with no escalation of care required.

d. Streamlined Workflow with Single-Use Bronchoscopes:

The use of two Ambu^®^ aScope™ 5 bronchoscopes (Ambu A/S, Ballerup, Denmark) simultaneously connected to a single monitor with split-screen mode allowed a compact setup that did not require an additional tower or imaging system. This portability may make the technique particularly appealing in lower-resource settings or satellite hospitals.

### 4.2. Limitations

Despite the strengths of this study, several limitations must be acknowledged:

- Single-Centre Design: The single-centre, retrospective design introduces the possibility of selection bias and reflects the experience of a team already familiar with advanced bronchoscopic techniques. The findings may reflect operator expertise and setup-specific advantages that may not generalize to other centres without comparable equipment or procedural expertise;
- Lack of Control Group: We did not compare our method directly to conventional bronchoscopy or CBCT-guided procedures. Future randomized or matched-cohort studies would help clarify comparative performance;
- Learning Curve and Technical Demands: Although our diagnostic yield was consistently high, an informal comparison between early and late cases revealed a trend toward shorter procedure times and improved efficiency in later cases, suggesting a learning curve effect. Operating two bronchoscopes simultaneously, navigating, coordinating instruments, and monitoring two video feeds requires training and procedural familiarity. The team-based nature of this approach also necessitates synchronized workflow and experienced support staff, larger datasets and multicentre validation are necessary to confirm reproducibility;
- Use of Rigid Bronchoscopy: Although rigid bronchoscopy provided airway stability and space for dual-scope access, this may not be feasible in centres lacking anaesthetic support or rigid instruments.

### 4.3. Clinical Implications

Given its high yield and safety profile, the dual-bronchoscope technique offers a pragmatic solution for centres seeking to improve peripheral lesion diagnosis without acquiring CBCT or robotic navigation systems.

Although CBCT-assisted bronchoscopy improves tool-in-lesion confirmation, it generally requires a hybrid OR/angiography suite, specialized staff, and (often) general anaesthesia, elements that increase fixed and variable costs and limit availability.

Single-use bronchoscopes can shift costs from reprocessing and repair as well as reduce cross-contamination risk. Several analyses and reviews suggest potential cost-consequence advantages vs. reusable scopes depending on local volumes and infection risk profiles [15,16].

In our study, the single-use bronchoscopes (Ambu^®^ aScope™ 5) required cost approximately EUR 300 each. This is substantially less than the capital and operating costs of CBCT, which requires investment exceeding EUR 250,000. Moreover, procedure duration of 42 min compares favourably with reported CBCT-guided procedures (65–80 min) [12,17]. Although formal cost-effectiveness modelling was not performed, these data suggest that the kissing probe technique may represent a financially accessible alternative for centres without advanced imaging and may serve as a bridge technology for interventional pulmonologists, wishing to expand their diagnostic capabilities with minimal new infrastructure.

Furthermore, the technique is scalable and adaptable across clinical settings and may be particularly beneficial in patients where transthoracic biopsy is contraindicated or carries a higher risk of pneumothorax.

## 5. Conclusions

The simultaneous use of dual bronchoscopes represents a practical and effective advancement in the bronchoscopic diagnosis of peripheral pulmonary lesions. In our retrospective study of 43 patients, this technique demonstrated a high diagnostic yield (83.7%) and excellent safety profile, with no major complications and a low incidence of manageable bleeding.

By combining real-time radial EBUS localization with cryobiopsy through an ultra-thin bronchoscope, this approach allows precise, tool-in-lesion tissue acquisition without the need for expensive navigation platforms or cone-beam CT. The additional advantage of immediate post-biopsy visualization and control of potential bleeding makes this a particularly attractive option for interventional pulmonologists operating in settings with limited access to advanced imaging technologies.

While promising, these findings warrant further validation through multicentre studies and direct comparison with existing modalities. Standardization of the technique, training pathways, and economic evaluation will also be important to support broader adoption.

In conclusion, the dual-bronchoscope approach offers a scalable, safe, and diagnostically powerful tool that may significantly enhance the bronchoscopic evaluation of peripheral lung lesions especially in resource-limited environments.

## Figures and Tables

**Table 1:** Patient Characteristics.

Patient Characteristics	Value (*N* = 43)
Age, mean (range)	64.3 years (38–82)
Sex (*n*, %)	26 male (60.5%), 17 female (39.5%)
Smoking history	21 current smokers (48.8%)
	15 former smokers (34.9%)
	7 never smokers (16.3%)
Symptomatology	Cough (72%)
	Weight loss (26%)
	Haemoptysis (14%)
Lesion size	Mean: 24.6 mm (range: 10–40 mm)
Lesion location	RUL (20), LUL (10), RLL (6), LLL (7)
Presence of bronchus sign in CT scan	36 patients (83.7%)

**Table 2:** Procedural Metrics.

Procedural Metrics	Result
Mean procedure time	42 ± 8 min
Mean number of cryobiopsy samples	3.1 per patient (range: 2–5)
Tool-contact confirmation (EBUS + fluoroscopy)	81.4% (35/43)
Radial probe ultrasound signal quality	Clear concentric in 23 cases (53.5%)
	Eccentric in 14 cases (32.5%)

**Table 3:** Histological Findings.

Histological Diagnosis	Number of Patients (*n* = 43)
Adenocarcinoma	18 (41.8%)
Squamous cell carcinoma	9 (20.9%)
Small cell lung carcinoma	2 (4.7%)
Atypical carcinoid	1 (2.3%)
Organizing pneumonia	4 (9.3%)
Granulomatous inflammation (TB)	2 (4.7%)
Nondiagnostic (non-specific tissue)	7 (16.3%)

**Table 4:** Complications.

Complications	Number of Patients	Severity/Management
Minor bleeding	9 (20.9%)	Controlled with suction/cold saline
Moderate bleeding	1 (2.3%)	Required temporary wedging, no epinephrine needed
Severe bleeding	0	-
Pneumothorax	0	-
Cryoprobe damage due to contact with radial probe	0	Prevented by systematic withdrawal before freezing

## Data Availability

Data may be made available from the corresponding author upon reasonable request.

## Supplementary Materials

The following supporting information can be downloaded at: https://www.mdpi.com/article/10.3390/jcm14186425/s1, Video S1: Single-operator PPL biopsy using “kissing probe” technique (step-by-step); Video S2: Dual-operator PPL biopsy using “kissing probe” technique (step-by-step).

## Abbreviations

The following abbreviations are used in this manuscript:

ENB	Electromagnetic navigation bronchoscopy
RP-EBUS	Radial probe endobronchial ultrasound
PPL	Peripheral pulmonary lesions
CBCT	Cone-beam computed tomography
IHC	Immunohistochemistry

## References

[1] Yankelevitz D.F., Yip R., Smith J.P., Liang M., Liu Y., Xu D.M., Salvatore M.M., Wolf A.S., Flores R.M., Henschke C.I. (2015). CT Screening for Lung Cancer: Nonsolid Nodules in Baseline and Annual Repeat Rounds. Radiology.

[2] Wang Memoli J.S., Nietert P.J., Silvestri G.A. (2012). Meta-analysis of guided bronchoscopy for the evaluation of the pulmonary nodule. Chest.

[3] Balasubramanian P., Abia-Trujillo D., Barrios-Ruiz A., Garza-Salas A., Koratala A., Chandra N.C., Yu Lee-Mateus A., Labarca G., Fernandez-Bussy S. (2024). Diagnostic yield and safety of diagnostic techniques for pulmonary lesions: Systematic review, meta-analysis and network meta-analysis. Eur. Respir. Rev..

[4] Verhoeven R.L.J., Kops S.E.P., Wijma I.N., Ter Woerds D.K.M., Van der Heijden E.H.F.M. (2023). Cone-beam CT in lung biopsy: A clinical practice review on lessons learned and future perspectives. Ann. Transl. Med..

[5] Sriprasart T., Aragaki A., Baughman R., Wikenheiser-Brokamp K., Khanna G., Tanase D., Kirschner M., Benzaquen S. (2017). A Single US Center Experience of Transbronchial Lung Cryobiopsy for Diagnosing Interstitial Lung Disease with a 2-Scope Technique. J. Bronchol. Interv. Pulmonol..

[6] Qi Q., Fang W., Yang L., Liu Y., Xu R., Liu D. (2025). Comparative diagnostic performance and safety of radial endobronchial ultrasound versus its combination with electromagnetic or virtual bronchoscopic navigation for peripheral pulmonary lesions: A retrospective study. Ther. Adv. Respir. Dis..

[7] Zhang Q., Wen F., Wu X., Yang H., Li X., Luo P., Liu H., Wang Z., Herth F.J.F., Zhang X. (2025). Shape sensing robotic assisted bronchoscopy versus virtual bronchoscopic navigation in the diagnosis of peripheral pulmonary nodules. Sci. Rep..

[8] DiBardino D.M., Kim R.Y., Cao Y., Andronov M., Lanfranco A.R., Haas A.R., Vachani A., Ma K.C., Hutchinson C.T. (2023). Diagnostic Yield of Cone-beam-Derived Augmented Fluoroscopy and Ultrathin Bronchoscopy Versus Conventional Navigational Bronchoscopy Techniques. J. Bronchol. Interv. Pulmonol..

[9] Lin C.K., Fan H.J., Yao Z.H., Lin Y.T., Wen Y.F., Wu S.G., Ho C.C. (2021). Cone-Beam Computed Tomography-Derived Augmented Fluoroscopy Improves the Diagnostic Yield of Endobronchial Ultrasound-Guided Transbronchial Biopsy for Peripheral Pulmonary Lesions. Diagnostics.

[10] Sobieszczyk M.J., Yuan Z., Li W., Krimsky W. (2018). Biopsy of peripheral lung nodules utilizing cone beam computer tomography with and without trans bronchial access tool: A retrospective analysis. J. Thorac. Dis..

[11] Kops S.E.P., Heus P., Korevaar D.A., Damen J.A.A., Idema D.L., Verhoeven R.L.J., Annema J.T., Hooft L., Van der Heijden E.H.F.M. (2023). Diagnostic yield and safety of navigation bronchoscopy: A systematic review and meta-analysis. Lung Cancer.

[12] Brown M.V., Badiei A., Arnold M., Jersmann H., Sullivan T., Fielding D., Nguyen P. (2024). The Diagnostic Yield of Cone Beam CT Combined With Radial-Endobronchial Ultrasound for the Diagnosis of Peripheral Pulmonary Nodules: Systematic Review and Meta-Analysis. CHEST Pulm..

[13] Lee J., Song J.U. (2023). Diagnostic yield of radial probe endobronchial ultrasonography-guided transbronchial biopsy without fluoroscopy in peripheral pulmonary lesions: A systematic review and meta-analysis. Thorac. Cancer.

[14] Kho S.S., Chan S.K., Yong M.C., Tie S.T. (2019). Performance of transbronchial cryobiopsy in eccentrically and adjacently orientated radial endobronchial ultrasound lesions. ERJ Open Res..

[15] Podder S., Perez O., Kivlin W., Singh H., Benn B., Kurman J. (2024). Role of single-use flexible bronchoscopes. AME Med. J..

[16] Kristensen A.E., Kurman J.S., Hogarth D.K., Sethi S., Sørensen S.S. (2023). Systematic Review and Cost-Consequence Analysis of Ambu aScope 5 Broncho Compared with Reusable Flexible Bronchoscopes: Insights from Two US University Hospitals and an Academic Institution. PharmacoEconomics-Open.

[17] Bhadra K., Setser R.M., Condra W., Bader B.A., David S. (2024). A Cone Beam CT Bronchoscopy Study of the Ultrathin Cryoprobe for Biopsy of Peripheral Lung Lesions. J. Bronchol. Interv. Pulmonol..

